# The effect of source expertise on the persuasiveness and sharing of health information on social media: A systematic review

**DOI:** 10.3758/s13423-026-02943-2

**Published:** 2026-06-15

**Authors:** Benjamin P. Simmonds, Keith J. Ransom, Emily Mullins, Rachel G. Stephens

**Affiliations:** 1 School of Psychology, College of Education, Behavioural and Social Sciences, Adelaide University, Hughes Building, North Terrace, Adelaide, SA 5000 Australia; 2 School of School of Computer Science and Information Technology, College of Engineering and Information Technology, Adelaide University, Adelaide, Australia

**Keywords:** Judgement and decision-making, Expertise, Health information, Social media

## Abstract

People increasingly rely on social media for health information, despite substantial variability in information quality. We conducted a systematic review and meta-analysis examining whether people are sensitive to source expertise when evaluating and sharing online health claims, and if this depends on whether the source is an individual expert (e.g., a physician) or an expert group (e.g., a health organisation). Three searches across four databases identified 25 individual studies published across 22 papers. From these studies, we found that people were only marginally more persuaded by health experts than nonexperts, and that this effect was not moderated by expert type (individual vs. group). However, we found tentative evidence that experts are more persuasive when accompanied by congruent credibility cues, but that expertise can backfire when these cues are incongruent. Evidence regarding people’s sensitivity to source expertise when sharing was mixed, reflecting heterogeneity in study designs. This review highlights several limitations in the current literature, indicating the need for further research that accounts for how both experts and nonexperts are perceived in online health contexts. We discuss implications and propose several avenues for future research and potential interventions.

## Introduction

Social media platforms are popular sources of health information, with one third of adults from the United States reporting that they turn to them at least some of the time (Pew Research Center, 2026). Yet health content posted to platforms such as Facebook, Instagram, X (formerly Twitter), YouTube, and TikTok is largely unregulated and varies widely in quality (Afful-Dadzie et al., 2023; Suarez-Lledo & Alvarez-Galvez, 2021). The sheer volume and often conflicting nature of online health information further compounds, this issue, making it difficult for users to evaluate what is true and what is false (Jiang, 2022; Pew Research Center, 2026). The consequences of this challenge became particularly evident during the COVID-19 pandemic, when the World Health Organization declared an “Infodemic”—noting that an overabundance of both accurate and inaccurate information threatened disease mitigation efforts (World Health Organization, 2020). Understanding what makes online health information persuasive, and what drives people to share it, is therefore of clear practical relevance to health communication research and practice, while also informing social-cognitive theories of belief updating and information transmission.

When evaluating the truthfulness of a claim, people often rely on the conclusions made by others to inform their judgements (Nowak et al., 2019; Ransom et al., 2021). On social media, however, sources can vary widely in their level of *expertise*—defined as the extent to which a person holds more true beliefs and fewer false beliefs concerning a given domain relative to most people (Goldman, 2018). To optimise their judgements, people should therefore generally weight expert sources higher than nonexpert sources; a tendency broadly observed in prior research on persuasion (Chaiken, 1980; Petty & Cacioppo, 1986; Pornpitakpan, 2004).

However, such weighting becomes more complicated when concerning matters of health because, unlike other domains of expertise (e.g., high energy particle physics, deep-sea biology, cryptology), nonexperts may be able to provide distinctly valuable information. More specifically, patients or carers—typically considered nonexperts—may acquire ‘lay expertise’ through experiential knowledge related to having or managing a health condition (Borkman, 1976; Prior, 2003). Such knowledge can be uniquely informative by providing pragmatic and individualised suggestions oftentimes distinct from the generalised, theory-based approaches taken by health professionals (Halloy et al., 2023). People appear to recognise the value in experiential knowledge, as evidenced by its general persuasiveness (Burgers et al., 2012), as well as by the popularity of health-related support groups, where people experiencing similar conditions can share experiential knowledge amongst themselves (Rueger et al., 2021). How people weigh experts against nonexperts in the health domain is therefore an important question to examine.

Social media platforms allow users to rapidly share or repost health information across large networks, which complicates experts’ influence because their persuasive impact depends partly on how widely their content is shared. However, the factors that drive sharing behaviour are unclear. If people share content that they believe to be true, as some research suggests they do (e.g., Ahmed & Rasul, 2022; Li & Sakamoto, 2014), then source expertise—a cue to information accuracy—should influence sharing behaviour. Yet, other research has identified a weaker link between sharing behaviour and perceived accuracy, with social motivators inherent to social media platforms—such as the desire to signal group membership—drawing attention away from concerns of accuracy (Epstein et al., 2023; Pennycook et al., 2021). Systematic investigation into how source expertise influences the sharing of health claims is therefore needed.

A potentially important aspect of expert influence concerns the type of expert source present. On social media, expert sources may take the form of *individual experts* (e.g., physicians) or *expert groups* (e.g., health institutions), raising the question of whether people consider and share information from these sources differently. One possibility is that people find expert groups to be more persuasive because they represent collectives of many individual experts (Simmonds et al., 2023), consistent with research suggesting that the number of sources supporting a claim is influential (e.g., Bond, 2005; Simmonds et al., 2024). However, people sometimes underweight aggregated opinions (Oktar & Lombrozo, 2026), at least partially due to the computational complexity of evaluating their informativeness (Oktar & Lombrozo, 2025) and the various ways in which the beliefs of a group may be conceptualised (Lackey, 2012). How people weigh individual experts relative to expert groups and, in turn, how this affects sharing behaviour is therefore an additional consideration that warrants investigation.

The current systematic review has three aims: to examine (1) how source expertise affects the persuasiveness of health claims, (2) how source expertise affects people’s online sharing behaviour, and (3) whether these effects differ for individual experts versus expert groups. We limit our scope to social media contexts. In addition to addressing these aims, we seek to critically evaluate how source expertise is conceptualised and operationalised across the existing literature. Furthermore, we will identify recurring interaction effects between expertise and other online cues and conclude by discussing the theoretical and practical implications of our review.

## Method

The registered protocol for this review is available on PROSPERO CRD42023482399, where we preregistered the aims, eligibility criteria, search strategies, and data synthesis methods. The aims outlined in this protocol were reworded slightly to improve clarity. This review follows the Preferred Reporting Items for Systematic reviews and Meta-Analyses (PRISMA) guidelines (Page et al., 2021).

### Eligibility criteria

#### Study design

We included quantitative studies employing correlational or experimental methods that directly compared outcomes across a nonexpert and expert level. Qualitative studies, grey literature, theses, reviews, meta-analyses, non-English language papers, and papers without full-text availability were excluded.

#### Population and setting

Since social media is widely used (Gottfried, 2024; Kemp, 2023) our target population was healthy adults from any country. We included studies using settings generalisable to real social media platforms, defined broadly as any online platform enabling the publishing and sharing of information between users. Therefore, settings included social media, blogs, forums, or Q&A platforms (e.g., Yahoo! Answers), as well as those that used simulated (e.g., Simmonds et al., 2023) or imagined (e.g., Sohn & Choi, 2019) social media environments.

#### Exposure

Eligible studies examined exposure to differing levels of source expertise, defined broadly as a combination of symbolic cues held by an individual or group that implies ready access to domain-specific evidence and the knowledge required to effectively evaluate that evidence. Expertise was categorised into three levels: nonexperts (i.e., general users, not including celebrities, politicians, or other sources that lack expertise but may be influential due to their authoritativeness or familiarity), individual experts (e.g., doctors, researchers), and expert groups (e.g., health institutions). Cues indicating expertise in social media settings included verification icons, credentials, institutional affiliations, and organisational logos. Studies were excluded if they did not directly compare experts (including either individual experts or expert groups) to nonexperts.

#### Outcome

We included studies that examined the persuasiveness and/or sharing of health claims across source expertise levels. Persuasiveness encompassed quantitative measures of attitudes, beliefs, or perceived source credibility. Sharing encompassed quantitative measures of the self-reported or estimated likelihood that a person would repost a health claim on social media, or—for studies using real-world social media data—actual repost counts.

#### Stimuli

Eligible studies examined the above outcomes with respect to health claims, encompassing topics such as physical activity, nutrition, substance use, immunisation, disease, injury, chronic conditions, sexual health, and mental health, among others. The actual truthfulness of the claim was not used as a criterion for eligibility. Studies using both health and nonhealth claims were eligible, but only data pertaining to the health claims were extracted.

### Information sources

We searched four online databases: PsycINFO (OVID interface), Scopus (Elsevier interface), Web of Science (Thomson Reuters), and Sociological Abstracts (ProQuest). An initial search was conducted in November 2023, limited to English-language journal articles and conference papers published from 2000 onwards. A second search in October 2024 and a third search in September 2025 identified more recent publications. We also searched reference lists of included papers to identify additional studies missed by our searches.

### Search strategy

We created four search strategies, one for each database. All strategies were structured around four keywords reflecting our inclusion criteria: Social Media (setting/population), Expertise (exposure/intervention), Influence (outcome, including both persuasion and sharing), and Health (stimuli). Under each keyword, we included terms that were either synonymous or conceptually related to the keyword. Strategies were formatted according to each database, and peer-reviewed by a research librarian prior to use.

### Selection process

Search results were imported into Covidence for screening. Covidence is a collaborative online tool that assists with finding and removing duplicates, screening papers and extracting relevant data. Prior to title and abstract screening, Author 1 and Author 3 conducted a calibration exercise with a sample of 10 papers to ensure consistency. They then independently screened the full set of titles and abstracts, resolving any disagreements amongst themselves. Papers included during this process continued to full text screening, which followed the same procedure. Papers that passed both screening stages were tagged based on their outcome measure (persuasion and/or sharing) and expertise type (individual and/or group) for subsequent analysis.

### Data collection process

Data were extracted by Author 1 using a standardised form that captured relevant information about each study (see Table 1 for a template). This included author names, affiliations, countries and contact information. We also recorded sample characteristics, such as the sample size, country, age distribution, and gender split. Furthermore, we extracted details about the study design, methodology, expert type (i.e., individual expert vs. expert group), and outcome measure(s) used. Finally, we wrote a brief narrative summary of the most relevant results, including interaction effects between expertise and other variables. Author 1 and Author 3 independently piloted the standardised form on five randomly selected studies, compared their results, and made minor adjustments to the form and to the extractions accordingly.

**Table 1:** Summary of all studies included in the final sample

Study	Population and design							Expertise		Measures		Results
Paper	Research design	Sample country	Sample size	Social media platform	Health topic	Age, mean (*SD*)	Female, %	Individual or group?	Definition	Persuasion	Sharing	Findings
Alvarez-Mon et al. (2021)	Observational	N/A	22,092 posts	Twitter	Antipsychotic medications	N/A	N/A	Individual and Group	*Nonexpert:* Patient, relative/friend of patient *Individual Expert:* Health professional *Expert Group:* Health institution	N/A	Retweet to tweet ratio	Posts by expert groups received more retweets than those by individual experts, who in turn received more than nonexperts
Borah and Xiao (2018) Study 1	Experimental	USA	340 participants	Facebook	Physical activity	19.80	66.20%	Group	*Nonexpert:* Ordinary user *Expert Group:* CDC	Credibility perceptions	N/A	Posts by expert groups were perceived as significantly more credible than those by nonexperts. Nonsignificant source expertise × ‘likes’ interaction effect
Borah and Xiao (2018) Study 2	Experimental	USA	552 participants	Facebook	Alcohol consumption	19.10	58.20%	Group	*Nonexpert:* Ordinary user *Expert Group:* WebMD	Credibility perceptions	N/A	Posts by expert groups were perceived as significantly more credible than those by nonexperts. Nonsignificant source expertise × ‘likes’ interaction effect
Carabot et al. (2023)	Observational	N/A	83,129 posts	Twitter	Paracetamol, tramadol, codeine	N/A	N/A	Individual and Group	*Nonexpert:* Patient, relative/friend of patient *Individual Expert:* Health professional *Expert Group:* Health institution	N/A	Retweet to tweet ratio	Posts by expert groups received more retweets than those by individual experts, who in turn received more than nonexperts
Harris et al. (2024)	Observational	N/A	7,720 accounts	Twitter	Vaccination	N/A	N/A	Individual	*Nonexpert:* Ordinary user *Individual Expert:* User holding indicators of health expertise	N/A	H-index of retweets across posts	Posts by individual experts received more retweets than those by nonexperts
Heiss et al. (2024)	Experimental	USA	956 participants	TikTok	Depression and suicide	40.33 (11.13)	44.00%	Individual	*Nonexpert:* Speaker wearing casual attire appearing in a bedroom setting *Individual Expert:* Speaker wearing business casual attire who identifies as a clinical psychologist	Misperceptions and perceived source and content credibility	N/A	Individual expert sources perceived as less credible than nonexpert sources. No significant difference in perceived content credibility. Including a scientific reference in their message did not increase the persuasiveness of individual experts
Jia et al. (2024)	Observational	N/A	362 posts	Twitter	COVID-19 vaccination	N/A	N/A	Individual	*Nonexpert:* Ordinary users *Individual Expert:* Health professional or public health expert	N/A	Number of retweets	No significant difference in number of retweets received by individual experts and nonexperts
Kareklas et al. (2015) Study 2	Experimental	USA	272 participants	Website comment section	Vaccination	34.40	48.60	Individual	*Nonexpert:* English literature student *Individual Expert:* Medical doctor	Source credibility	N/A	Individual experts perceived as significantly more credible than nonexperts
Kothari et al. (2022)	Observational	N/A	1,253 posts	Twitter	COVID-19	N/A	N/A	Individual and Group	*Nonexpert:* Citizen *Individual Expert:* Health professional or researcher *Expert Groups:* Gov. agency or NGO	N/A	Number of retweets	Posts by nonexperts received more retweets than those by individual experts or expert groups
Lee and Sundar (2013)	Experimental	USA	63 participants	Twitter	Weight loss	22.47 (0.67)	73.00	Individual	*Nonexpert:* Ordinary user *Individual Expert:* Medical doctor	Perceived content credibility	Behaviour intentions, including the likelihood of retweeting	No significant difference in persuasion or sharing behaviour between nonexperts and individual experts. Expert tweets were more persuasive and shared more than nonexpert tweets when follower counts were high; nonexpert tweets more persuasive and shared more than expert tweets when follower counts were low
Li et al. (2024)	Experimental	USA	873 participants	TikTok	COVID-19 vaccines and infertility	29.62 (6.57)	75.80	Individual	*Nonexpert:* Ordinary user *Individual Expert:* Medical expert or healthcare professional	Misbelief acceptance	N/A	Individual experts significantly more persuasive than nonexperts
Mao et al. (2024)	Observational	N/A	111 posts	TikTok	Acute pancreatitis	N/A	N/A	Individual	*Nonexpert:* Ordinary user *Individual Expert:* Medical doctor	N/A	Number of shares	No difference in the number of shares between individual experts and nonexperts
Ming et al. (2023)	Observational	N/A	168 posts	TikTok	Myopia	N/A	N/A	Individual and group	*Nonexpert:* Ordinary user *Individual Expert:* Health professional *Expert Group:* Academic institution	N/A	Number of shares	Posts by nonexperts received more shares than those by individual experts or expert groups
Poorisat et al. (2019)	Experimental	Thailand	499 participants	Discussion board	HIV symptoms and transmission	20.07 (1.37)	55.30	Individual	*Nonexpert:* Ordinary user *Individual Expert:* Medical doctor	Perceived message credibility	Behavioural intentions, including the likelihood of forwarding	No difference in persuasion or sharing behaviour between nonexperts and individual experts
Sharon et al. (2020) Study 2	Observational	N/A	2,583 answers	Yahoo! Answers	Vaccination	N/A	N/A	Individual	*Nonexpert*: Parent or ordinary user *Individual Expert:* Health professional	Whether answer was marked as the “Best Answer”	N/A	Answers by individual experts were more likely to be selected as the “Best Answer” compared to those by nonexperts
Sharon et al. (2020) Study 3	Experimental	USA	694 participants	Yahoo! Answers	Vaccination	Not Known	Not Known	Individual	*Nonexpert*: Parent or ordinary user *Individual Expert:* Health professional	Vaccine attitudes and source trustworthiness	N/A	Individual experts perceived as more trustworthy than nonexperts. This effect was stronger when the source was pro- rather than anti-vaccination
Simmonds et al. (2023) Study 1	Experimental	Mostly USA	103 participants	Twitter	Various topics	38.00	33.98	Individual and group	*Nonexpert:* Ordinary user *Individual Expert:* Medical doctor *Expert Group:* Health institution	Belief change	N/A	Both individual experts and expert groups were more persuasive than nonexperts. No significant difference between the persuasiveness of individual experts and expert groups
Simmonds et al. (2023) Study 2	Experimental	Mostly USA	101 participants	Twitter	Various topics	37.91	31.68	Individual	*Nonexpert:* Ordinary user *Individual Expert:* Medical doctor	Belief change	N/A	Individual experts more persuasive than nonexperts
Simmonds et al. (2024)	Experimental	Mostly USA	99 participants	Twitter	Various topics	40.52 (10.70)	41.41	Group	*Nonexpert:* Ordinary user *Expert Group:* Health institution	Belief change	N/A	Expert groups more persuasive than nonexperts. No significant interaction effect between expertise and consensus quantity
Sohn and Choi (2019)	Experimental	South Korea	256 participants	Facebook	Coffee powder as skin care	Not Known	57.42	Group	*Nonexpert:* Ordinary user *Expert Group:* University research laboratory	Perceived information credibility	Sharing intention	Expert groups more persuasive than nonexperts. No significant difference in sharing intention
Terada et al. (2023)	Observational	N/A	3,623 posts	Twitter	HPV vaccine	N/A	N/A	Individual	*Nonexpert:* Ordinary users *Individual Expert:* Health professional or researcher	N/A	Number of retweets	Posts by individual experts received more retweets than those by nonexperts
Vraga and Bode (2017)	Experimental	USA	1,384 participants	Twitter	Zika virus	19.40 (2.97)	52.90	Group	*Nonexpert:* Ordinary user *Expert Group:* CDC	Change in Zika misperceptions	N/A	Expert groups more persuasive than nonexperts
Walther et al. (2016)	Experimental	USA	354 participants	Yahoo! Answers	HIV	20.00	75.00	Individual	*Nonexpert:* Ordinary user *Individual Expert:* Previously worked with a professor in medicine	Perceptions of advice quality and effectiveness	N/A	No significant difference in persuasiveness of nonexperts and individual experts. Significant expertise × advice feasibility interaction effect
Wu et al. (2024)	Observational	N/A	412 posts	TikTok	Vaping cessation	N/A	N/A	Individual	*Nonexpert:* Ordinary user *Individual Expert:* Health professional	N/A	Number of shares	No difference in number of shares received by nonexpert and individual expert posts
Yang et al. (2024)	Observational	N/A	216 posts	TikTok	COVID-19 vaccination	N/A	N/A	Individual	*Nonexpert:* Ordinary user *Individual Expert:* Health care provider or public health scientist	N/A	Number of shares	Posts by individual experts received more shares than those by nonexperts

### Study risk of bias assessment

Study quality was assessed using an adapted version of the QualSyst quality assessment tool, which is suitable for a variety of research designs (Kmet et al., 2004). This tool considers multiple potential sources of bias, including in study design, participant selection, randomisation, outcome measurement, sample size, data analysis, and result reporting. Raters scored each criterion in terms of how well the study met certain quality requirements on a scale with three points: Yes (2), Partial (1), and No (0). A total quality assessment score was generated by adding up the criterion scores, ignoring N/As. Studies that scored below 75% were excluded. To pilot the tool, Author 1 and Author 3 independently scored five randomly selected studies. Interrater reliability was calculated using Cohen’s weighted kappa, which produced a value of.44, indicating moderate^1^ levels of agreement between the two reviewers. Reviewers discussed any discrepancies in order to improve consistency, and the first reviewer completed the remaining assessments.

### Synthesis methods

To synthesise results comparing the persuasiveness of health claims across levels of source expertise, we conducted a random effects meta-analysis. If exact effect sizes were not available, we estimated them based on the available data. Two relevant comparisons (Sharon et al., 2020; Vraga & Bode, 2017) were excluded from the meta-analysis due to insufficient data. We also conducted a moderation analysis to determine whether the effect differed based on whether the comparison involved individual experts or expert groups.

Due to sizable heterogeneity in study design, we synthesised results regarding sharing behaviour using a narrative synthesis supplemented by content analysis (following the method outlined by Nicmanis, 2024). This process involved the first author iteratively coding relevant parts of each paper in NVivo and progressively refining and structuring these codes into categories and subcategories to devise a final analytical structure. A similar procedure was used to synthesise interaction effects and to examine common operationalisations of source expertise across papers.

## Results

### Study selection

**Fig. 1:**
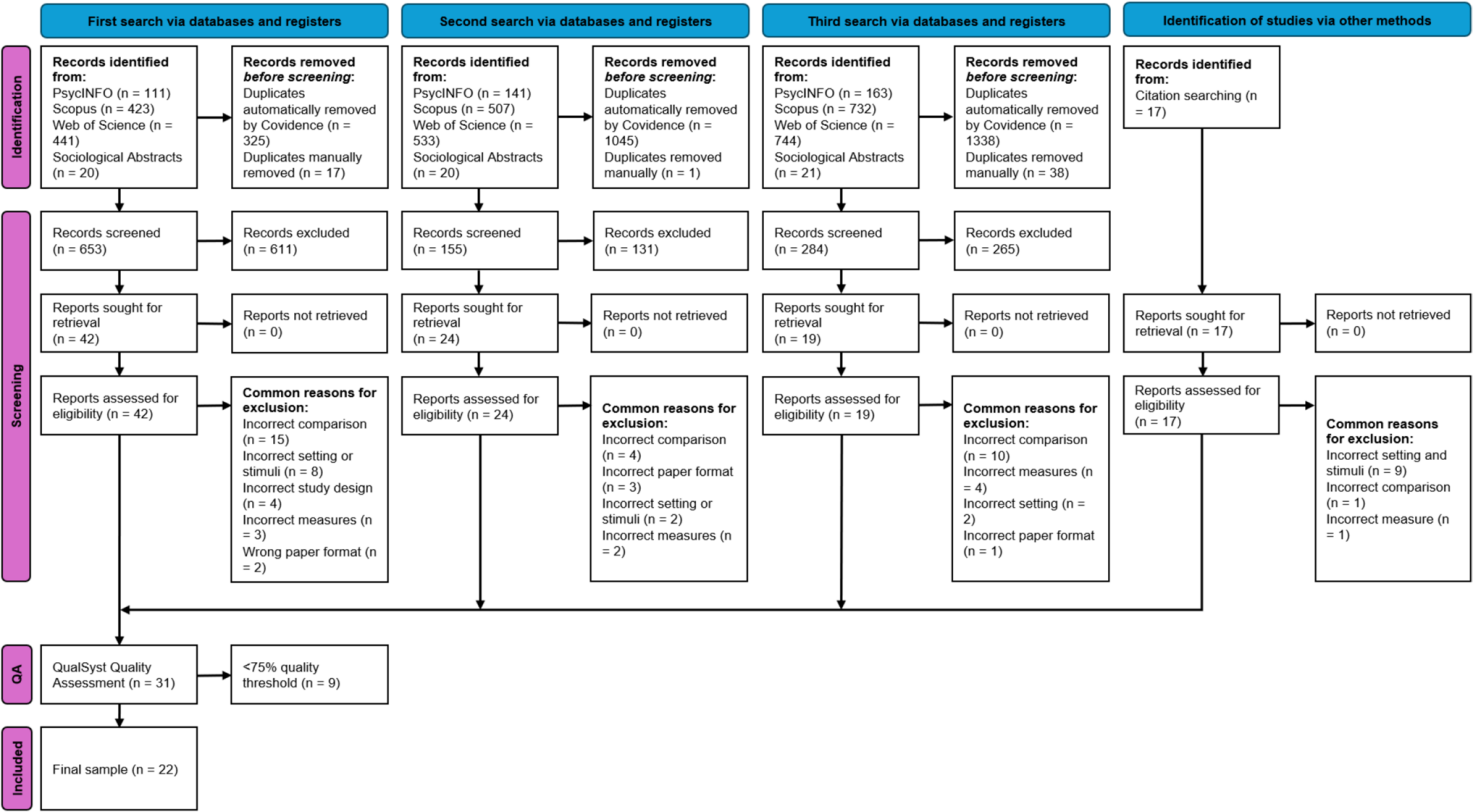
PRISMA flow diagram

Figure 1 illustrates the selection process using a PRISMA flow chart. From this point onwards, we use the term *paper* to refer to a single journal article and use the term *study* to refer to a single investigation which includes at least one predictor and outcome; importantly, a single paper may contain multiple studies. Our first search identified 995 papers: 111 from PsycINFO, 423 from Scopus, 441 from Web of Science, and 20 from Sociological Abstracts. Covidence automatically removed 325 duplicates, and 17 additional duplicates were removed manually. Author 1 and Author 3 completed title and abstract screening for 653 papers, where they excluded 611. These authors then completed full-text screening for the remaining 42 papers, where they excluded a further 32.

Our second search, conducted approximately 1 year later, identified 1,201 papers: 141 from PsycINFO, 507 from Scopus, 533 from Web of Science, and 20 from Sociological Abstracts. Covidence automatically removed 1,045 duplicates and one duplicate was removed manually, leaving a sample of 155 papers. Given the high level of agreement between reviewers in the first search, only Author 1 screened for inclusion, excluding 131 papers during title and abstract screening, and a further 11 during full-text screening.

Finally, our third search, conducted another year later, identified 1,660 papers: 163 from PsycInfo, 732 from Scopus, 744 from Web of Science, and 21 from Sociological Abstracts. A total of 1,338 duplicates were automatically removed and 38 were manually removed. Author 1 completed title and abstract screening of the remaining 284, sending 19 to full-text screening, where a further 17 were excluded.

We searched for additional relevant papers by scanning the reference lists of all 63 papers that passed title and abstract screening. This yielded an additional 17 papers, of which 11 were later excluded during screening.

### Quality assessment

We assessed the quality of 31 papers that made it through title/abstract and full-text screening using the QualSyst quality assessment tool. Nine that scored below the 75% score threshold were excluded.^2^ The following analyses were conducted on the remaining 22 papers.

### Study characteristics

The final sample consisted of 25 studies across 22 papers (see Table 1). Fourteen studies (56%) were experimental, where the expertise of the source was under the direct control of the experimenters. All experimental studies included a measure of persuasion and three included a measure of sharing. Nine used messages from individual experts, six used messages from expert groups, and one used messages from both. Participants in these studies were predominantly recruited from the United States (12/14). The remaining two studies recruited participants from Thailand and South Korea, respectively.

Eleven (44%) studies were observational, meaning that they analysed real-world social media data and therefore could not directly manipulate the expertise of the source. The majority (10/11) of these studies measured sharing. The remaining study measured the persuasiveness of health claims by examining whether it was marked as the “Best Answer” on Yahoo! Answers. All observational studies included messages from individual experts, and four also included messages from expert groups.

Studies in our sample covered a broad range of health topics and social media platforms. The most common topics were vaccination (8/25) and COVID-19 (4/25), but other topics included weight loss, mental health, disease transmission, and medicine use. Common social media platforms included: X/Twitter (11/25), TikTok (6/25), Facebook (3/25), and Yahoo! Answers (3/14).

### Syntheses of finding

#### Source expertise: Operationalisations and theoretical frameworks

Prior to addressing our research aims, we examined cross-paper heterogeneity in how source expertise was operationalised and measured. Given that definitions of expertise vary broadly (Croce & Baghramian, 2024), and perceptions of who is considered an expert likely differ across cultures and individuals (Germain & Enrique Ruiz, 2009; Yuan et al., 2019), it is important to clarify the prevailing conceptions of source expertise used in our sample.

We first examined how different types of sources were categorised as being either experts (including individual experts and expert groups) or nonexperts. Individual experts predominantly included health professionals such as physicians, medical scientists, and nurses. Expert groups included health and academic institutions, such as the World Health Organization (WHO) and the Centers for Disease Control and Prevention (CDC). Nonexperts, on the other hand, primarily comprised general social media users with no visible indicators of expertise, although this occasionally included students or patients and their friends or family members.

These categorisations impacted how expertise was signalled to the participant or identified by the researchers. A common thread across all expertise types was an emphasis on source cues (e.g., display names, handles, avatars, verification icons) as primary indicators of expertise. Nonexperts, on the other hand, were typically signalled through the absence of such cues. Notably, message-related cues were largely overlooked as potential indicators of expertise, with one exception (Li et al., 2024), where expertise was conveyed through the use of scientific rather than anecdotal evidence.

A particularly notable finding was that our sample drew upon a wide variety of general theoretical frameworks when examining expertise. The most common was based on work by Hovland et al. (1953), which frames expertise as a component of source credibility alongside trustworthiness. Under this framework, expertise is defined as “the extent to which a communicator is perceived to be a source of valid assertions” (p. 21), and is made distinct from trustworthiness, which is defined as “the degree of confidence in the communicator’s intent to communicate the assertions he considers most valid” (p. 21). Other research instead used dual-processing models of persuasion such as the Heuristic-Systematic Model (HSM) of information processing, where source expertise is a heuristic cue used when motivation and/or ability to systematically evaluate the truthfulness of a claim is low (Chaiken, 1980). Other framings included expertise as a cue to *authority* (e.g., Lee & Sundar, 2013)—which frames the influence of experts as one based in institutionalised power (Munduate & Medina, 2004)—and as a cue to *prestige* (Harris et al., 2024)—which frames the influence of experts as deriving from their social status or reputation (Henrich & Gil-White, 2001).

These framings are not necessarily contradictory, but they imply different underlying pathways through which experts gain their influence. Hovland et al.’s (1953) framework implies that people defer to expert opinion because they see experts as providing more valid claims. The HSM framework is similar but suggests that deference to experts often occurs because expertise is used as a simple heuristic that bypasses systematic considerations of argument quality. Prestige framings offer a third pathway by framing deference as a process which may be motivated by the desire to accrue social rank from high-prestige individuals, which includes experts (Henrich & Gil-White, 2001). Finally, authority framings suggest that this deference may occur through real or imagined social pressure.

Notably, the papers presented limited consideration of how expertise may uniquely interact with the health domain (e.g., the concept of the *lay expert*) or how it may uniquely manifest on social media (e.g., on the basis of source cues specific to social media, such as verification badges). The degree of heterogeneity in how source expertise is operationalised and framed is important to consider as we begin to address our research aims.

#### Effect of source expertise on persuasion

**Fig. 2:**
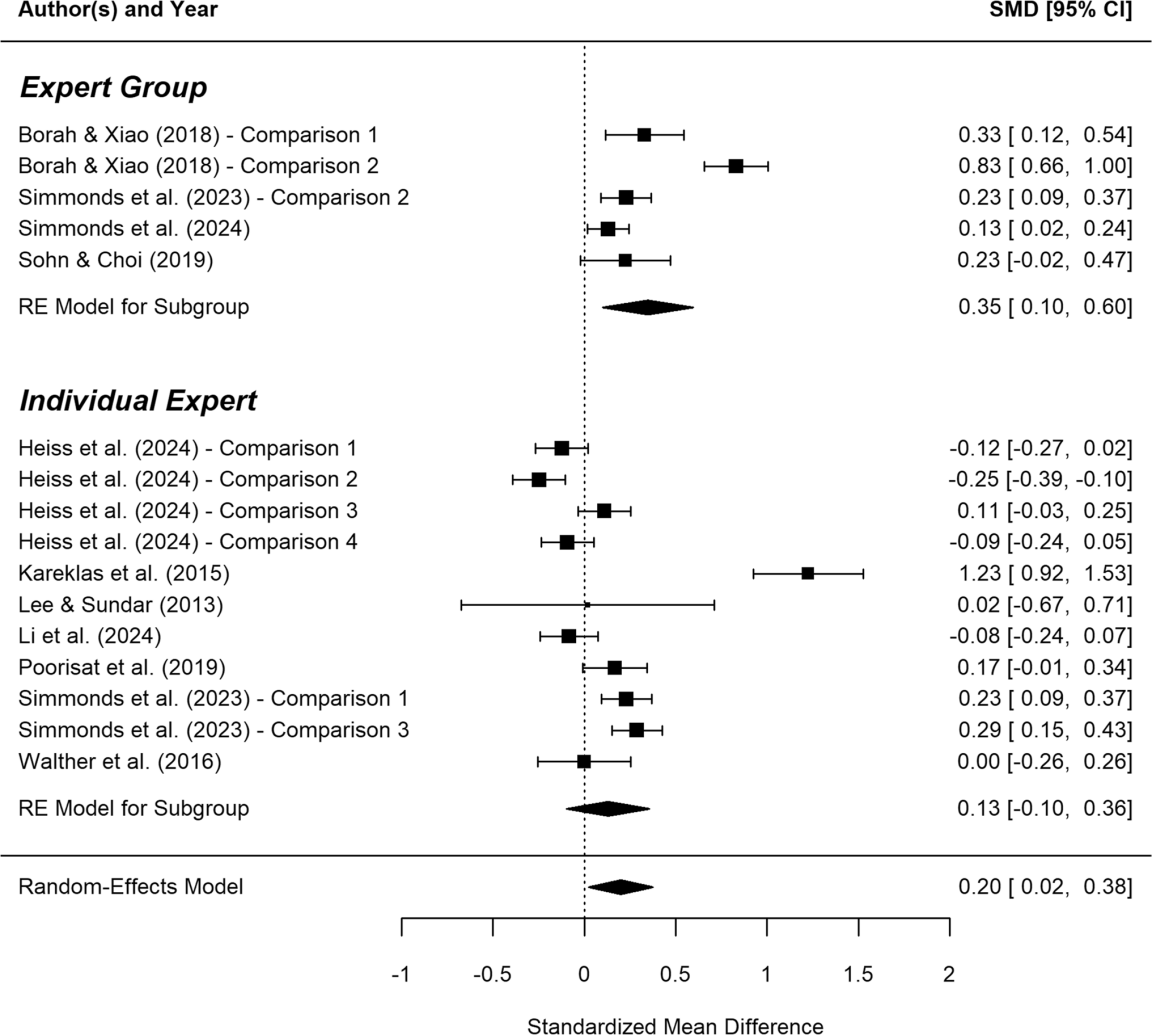
Forest plot displaying 16 effect sizes related to the effect of source expertise on persuasion. Effects are separated by expert type

To examine how source expertise affects the persuasiveness of health claims, we conducted a random effects meta-analysis. We limited this analysis to experimental papers to allow for a more robust comparison, ultimately including 16 estimated effect sizes from 10 papers. The analysis indicated that there was a small but significant effect of expertise on the persuasiveness of health claims (*g* =.20, *p* =.041, 95% CI [.009,.387]; see Fig. 2 for forest plot). However, there was significant heterogeneity in the sample, *Q*(14) = 180.57, *p* <.001, *I*^2^ = 94.73%, *τ*^2^ =.13, which may have been a result of the aforementioned variance in research methodology and in the operationalisation of source expertise.

Next, we were interested in examining whether this effect was moderated by the type of expert source viewed; that is, whether the participant encountered an individual expert or an expert group. A moderation analysis indicated that there was no significant difference in the effect of source expertise on the persuasiveness of health claims across expert source type, *Q*_M_(1) = 1.34, *p* =.246.

**Fig. 3:**
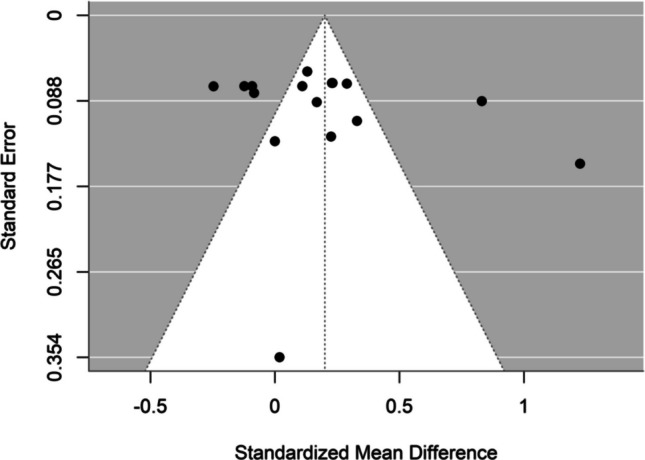
Funnel plot displaying good symmetry and thus low chance of publication bias

Finally, we examined whether the above results may have been influenced by publication bias. Visual examination of a funnel plot (Fig. 3) indicated good symmetry, and Egger’s regression test found no significant asymmetry (*b* =.09, *z* =.64, *p* =.525). We therefore deemed it unlikely that publication bias influenced the estimated effect size. In summary, we concluded that there is a small but significant effect of source expertise on the persuasiveness of health claims, regardless of the type of expert source viewed.

##### Interaction effects

To account for nuances not captured by our meta-analysis, we coded the results of all two-way interaction effects involving source expertise. A recurring pattern was what we term a *congruence* effect, where several studies examined how alignment between source expertise and other credibility cues moderated the persuasiveness of health claims. Congruence occurred when experts were paired with cues that similarly signalled high credibility. This would include, for example, an expert providing high-quality (rather than low-quality) advice, since advice quality is positively associated with source credibility (Feng & MacGeorge, 2010).

Across four relevant papers, two found a significant congruence effect, such that congruence further augmented persuasiveness. More specifically, health experts were most persuasive when holding high rather than low follower counts (Lee & Sundar, 2013), and when providing high-quality rather than low-quality advice (e.g., directing the participant towards immediately available anonymous HIV testing providers rather than directing towards an unavailable HIV testing smartphone application; Walther et al., 2016). Conversely, incongruence—such as when experts provided low quality advice or had a low follower count—led to a ‘backfire’ effect, where messages from health experts were perceived as being less persuasive than comparable messages from nonexperts.

However, this effect was not necessarily consistent. Borah and Xiao (2018) tested for a two-way interaction between source expertise and social endorsement (i.e., the number of likes received by a message) in two separate studies, finding nonsignificant effects in both instances. Similarly, Heiss et al. (2024) found that the persuasiveness of expert sources did not differ depending on if they cited a scientific source. Overall, these findings offer tentative evidence that people consider the congruence between source expertise and other credibility cues when online, though further research is needed.

#### Effect of source expertise on sharing behaviour

Studies examining sharing behaviour were more heterogeneous in design than those examining persuasion, making it difficult to justify conducting a meta-analysis. Instead, we conducted a content analysis, coding the results of 17 pairwise comparisons (expert vs. nonexpert) across 13 papers. Seven comparisons (41.18%) from five papers found greater sharing of expert posts, four comparisons (23.53%) from two papers found greater sharing of nonexpert posts, and six comparisons (35.29%) from six papers found no differences according to expertise. Thus, there was no discernible pattern regarding a main effect of source expertise on sharing behaviour in our sample.

**Fig. 4:**
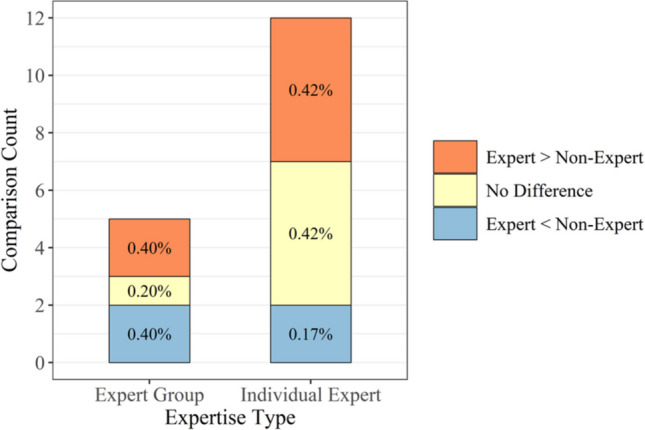
Stacked bar plot displaying the number of comparisons between expert and nonexpert sources that produced each type of effect on sharing behaviour. Effects are separated by expert type (expert groups vs. individual expert). (Colour figure online)

We next examined whether this effect differed by expert type (see Fig. 4). Of the 12 comparisons made between individual experts and nonexperts, five (41.67%) found greater sharing of individual expert posts, two (16.67%) found greater sharing of nonexpert posts, and five (41.67%) found no difference. Of the five comparisons between expert groups and nonexperts, two found greater sharing of expert group posts, two found greater sharing of nonexpert posts, and one found no difference. Thus, much like the broader effect on sharing behaviour, there was no clear pattern of results when separated according to expert source type.

## Discussion

Considering the common use of social media as a source of health information and the variable quality of that information, this review examined the effect of source expertise on the persuasiveness and sharing of online health claims, and whether this effect differed between individual experts and expert groups. Across three searches of four databases, we found that source expertise has a small, variable effect on persuasion, with a slight tendency to favour experts over nonexperts. While somewhat consistent with broader persuasion findings (e.g., Chaiken, 1980; Petty & Cacioppo, 1986; Pornpitakpan, 2004), the small effect size was unexpected given that experts are generally more informative than nonexperts (Ballantyne, 2019; Goldman, 2018).

One possible explanation is that nonexperts’ experiential knowledge bolsters their persuasiveness, aligning with prior research on ‘lay expertise’ (e.g., Halloy et al., 2023). However, studies in our meta-analysis were unable to directly test this explanation as they typically held message content constant and manipulated source cues alone (e.g., display names, handles, avatars, verification icons). A stronger test would allow evidence type to vary naturalistically with expertise, such as having experts provide scientific evidence and nonexperts provide anecdotal evidence.

An alternative explanation, however, is that people are sceptical towards experts on social media, perhaps because indicators of expertise online can be unreliable (Goga et al., 2015). This possibility would also explain why we found some evidence of a congruence effect, where health experts were most persuasive when paired with corroborating credibility cues—such as high follower counts (Lee & Sundar, 2013) and high quality advice (Walther et al., 2016)—but less persuasive than nonexperts when those cues instead implied low credibility. These effects suggest that people may consider additional indicators of credibility in order to ‘verify’ the legitimacy of experts online, although current evidence is limited.

Another key finding was that expert type (i.e., individual expert vs. expert group) did not significantly moderate the main effect of expertise on persuasion. This finding is somewhat at odds with our aforementioned scepticism account, since we might expect differences in scepticism depending on the type of expert source encountered. For example, it may be reasonable to be sceptical of an unfamiliar individual expert, but arguably less so towards an established expert group such as the CDC or WHO. Therefore, a better explanation for our findings may simply be inattention towards expertise. Such an account aligns with broader explanations for the sharing of misinformation online (e.g., Pennycook et al., 2021), and prior research that also identified a general lack of attentiveness towards other cues that can affect the informativeness of others’ conclusions, such as evidential diversity or source independence (e.g., Ransom et al., 2021; Yousif et al., 2019). A limitation of this account, however, is that it is unclear how inattention would give rise to a congruence effect, should such an effect be supported by further evidence.

Regardless, if our findings regarding persuasion reflect an inattentiveness to expertise, this inattentiveness appears to be further reflected in sharing behaviour. Results comparing people’s sensitivity with expertise when making sharing decisions were mixed, even when grouping based on expert type. This result may be explained by Pennycook et al.’s (2021) inattention account for misinformation sharing, which suggests that people are often distracted away from considering source credibility and instead base their sharing decisions on a range of other factors (Epstein et al., 2023). In line with this explanation, two studies from our sample (Jia et al., 2024; Ming et al., 2023) specifically criticised real expert-authored posts for being poorly designed, overly technical, and unengaging, potentially limiting their shareability. However, research in this area was predominantly observational, preventing causal inference.

A particularly notable limitation of the current literature is the lack of explicit consideration given to nonexpert credibility. Studies largely adopted a simple expert/nonexpert distinction, overlooking the possibility that nonexperts may be perceived as knowledgeable in their own right by accumulating relevant and uniquely informative experiential knowledge (Borkman, 1976; Dumez & L’Espérance, 2024). This style of conceptualisation risks limiting our understanding of why nonexpert voices can sometimes be so compelling to the general public.

Relatedly, studies often operationalised expertise in terms of the presence or absence of primarily source-related cues (e.g., verification icons, credentials, logos). This approach neglects the possibility that expertise may also be signalled through message-related cues, such as the types of evidence or arguments presented. Future research could draw on the sampling assumptions literature, which examines how people’s inferences about the evidence available to different sources shapes their belief revision (e.g., Simmonds et al., 2024). Experiments that take this approach could both motivate and constrain the development of formal computational models that can then be subjected to rigorous empirical testing.

### Additional key directions for future research

Based on the current state of the literature, we want this review to be a call for more research, and we propose several directions. First, there is a preponderance of observational social media research that, while extremely valuable for examining naturalistic behaviour, is limited in its ability to identify the causal mechanisms underpinning sharing behaviour. There is room for future research that attempts to combine the ecological validity of observational work with the control of experimental work. A good recent example of this approach is demonstrated by Alister et al. (2025), who observed participant behaviour in a realistic but isolated and controlled social media environment.

Next, our meta-analysis indicated that there was significant unexplained heterogeneity in expertise effects across studies, which may have partially stemmed from the high variability in health topics used between studies. Furthermore, many studies measured their outcome variables in respect to a single health topic or claim. This is a reasonable approach if it aligns with a specific defined scope, but future research aiming to identify more general expertise effects should use a broader sample of health-related topics.

Finally, the highly dynamic nature of social media policy and design is an important consideration for future research in this area. In 2022, X changed its verification system from a system designed to verify the identities of notable accounts, including health organisations and particularly prominent health experts, to a paid subscription service available to any user with a confirmed phone number (Twitter, 2022). This change led to several cases of people impersonating celebrities, corporations, and organisations (The Associated Press, 2023). While new icons have since been introduced to X to verify certain organisations and government-affiliated accounts (Traughber, 2022), the original verified icon is no longer a reliable indicator of an account’s identity, let alone their level of expertise. Furthermore, the advent of generative artificial intelligence and its rapid advancement presents a challenge for people to not just assess whether a health claim is true, but also whether it represents the opinion of a real person—a task that people often struggle with (Kenny et al., 2024). This distinction is important because fake social media accounts may be used by malicious actors to spread misinformation and create an impression of wide public support for certain beliefs (Ferrara et al., 2016). How people infer expertise in such an environment is, we believe, a pressing direction for future research.

### Applied implications

Our review has several important implications for practitioners that engage in health promotion activities on social media. First, since health experts appear to be only slightly more persuasive than ordinary social media users, the presence of experts alone may not be sufficient in shifting public opinion against opposing nonexperts. If this effect stems from inattention, then simple nudge interventions prompting consideration of expertise may help—much like how similar accuracy nudges can improve truth discernment (e.g., Pennycook et al., 2021). However, if this effect stems from scepticism, then more targeted interventions to reduce scepticism may be required.

Regardless of whether the effect stems from inattention or scepticism, a promising alternative approach may be to simply pair expert signals with congruent credibility cues. More specifically, health experts should aim to maximise their follower counts (Lee & Sundar, 2013) and focus on providing feasible and practical health advice (Walther et al., 2016), all while ensuring that more salient indicators of expertise (e.g., credentials, affiliations, logos) are highly visible. Furthermore, based on observations by Jia et al. (2024) and Ming et al. (2023), health experts should aim to improve the design, appeal, and relatability of their social media content by avoiding overly technical language and lecture-like formatting, both of which limit their shareability.

Finally, although we found limited evidence that people evaluate and share health information differently depending on whether the source is an individual expert or an expert group, the relative advantages of each remain worth recognising. Individual experts can often respond more rapidly to emerging public health crises without the delays of group coordination, and they may be better positioned to meet demands for relatable content. However, they also risk providing narrower perspectives potentially influenced by idiosyncratic biases. Expert groups, by contrast, can represent a broader array of opinions, access greater resources to operate at a larger scale, and serve as a convenient aggregator of the expert consensus. A limitation of these sources, however, is that their informativeness can be difficult to evaluate, especially when the group’s internal decision-making processes are unclear (Oktar & Lombrozo, 2025). Future research should further explore the unique advantages and disadvantages that different types of expert sources can offer.

### Limitations

We note that there are several potential limitations associated with the current work’s method and sample. First, as discussed, a substantial proportion of our sample—particularly those examining sharing behaviour—used observational designs for which causal inference was not possible. While such studies offer benefits in that they capture naturalistic behaviour in online environments, they cannot adequately control for confounding variables such as message content, follower counts, and platforms’ content algorithms.

Second, the sampling methods that these studies used were often unclear or potentially biased. In particular, a common approach involved searching posts by hashtag, sorting the results in descending order by likes or views, and sampling a limited number from the top (e.g., Ming et al., 2023; Yang et al., 2024). This approach has the benefit of filtering out largely unseen and uninfluential content but presents an unrepresentative picture of the type of content being posted to these platforms.

Finally, there were some potential limitations associated with the review’s exclusion criteria and quality assessment tool. The search strategy was limited to quantitative peer-reviewed journal articles indexed in four academic databases, which helped prioritise high-quality scientific articles, but narrowed the scope of the review. Additionally, the QualSyst quality assessment tool, although quantitative and flexible, remains largely subjective, even when using multiple reviewers. The tool also tends to disadvantage observational studies, which often scored lower on criteria related to the control of confounds and the reporting of subject characteristics. Nonetheless, we carefully considered these limitations and believe that our quality assessments were applied fairly and consistently.

## Conclusion

Amid concerns about the quality of health information available on social media, the current review examined how source expertise affects the persuasiveness and sharing of health claims online. After three systematic searches of four databases, we found a small but significant effect of source expertise on persuasion which was not moderated by whether the source was an individual expert (e.g., physician) or an expert group (e.g., health institution). However, we found tentative evidence that persuasion is moderated by the presence of additional congruent credibility cues, consistent with the notion that people are either sceptical or inattentive towards simple source expertise cues. Findings regarding sharing behaviour were considerably more mixed, likely reflecting substantial methodological heterogeneity across studies. Given the current state of the literature, there is a need for future research to explore the unique assumptions people make when responding to health experts online. Our findings imply that experts may struggle to maintain a persuasive advantage over nonexperts on social media. To maximise their impact, we recommend that health experts leverage additional indicators of source credibility to ‘verify’ their expertise, and work towards making their content more appealing and ultimately more shareable.

## Data Availability

Data and materials are available on the Open Science Framework: https://osf.io/s87j4/?view_only=b6637cacaa32419dafb890f3f3c7f912.

## Footnotes

1 Landis and Koch (1977) suggested that values between.41 and.60 indicated moderate rates of agreement beyond what might be expected by chance.

2 See OSF (https://osf.io/s87j4/overview?view_only=b6637cacaa32419dafb890f3f3c7f912) for full scoring details.

## Code availability

Code used to analyse data is available on the Open Science Framework: https://osf.io/s87j4/?view_only=b6637cacaa32419dafb890f3f3c7f912.

## Declarations

### Open practices statement

The data and materials for the review are available at: https://osf.io/s87j4/?view_only=b6637cacaa32419dafb890f3f3c7f912. The registered protocol for this review can be found on PROSPERO CRD42023482399.
